# Unilateral visual impairment in a patient undergoing chemotherapy: a case report and clinical findings

**DOI:** 10.1186/s12886-019-1246-3

**Published:** 2019-11-21

**Authors:** Xia Yuan, Yuliang Feng, Dan Li, Mei Li

**Affiliations:** 1 Department of Medical Oncology, Cancer Center, West China Hospital, Sichuan University, 37 Guoxue Alley, Chengdu, 610041 People’s Republic of China; 2 Department of Ophthalmology, West China Hospital, Sichuan University, Chengdu, 610041 China; 3 Department of Respiratory and Critical Care Medicine, West China Hospital, Sichuan University, Chengdu, 610041 China

**Keywords:** Vision loss, Chemotherapy, Nonarteritic anterior ischemic optic neuropathy

## Abstract

**Background:**

Visual impairment occurred as an infrequent form of chemotherapeutic toxicity and was often underestimated despite of several reports. We described a case of acute unilateral visual impairment after one cycle of intravenous chemotherapy of a normal dose, aiming at raising attention to chemotherapy-induced ocular toxicity.

**Case presentation:**

The patient developed a progressive vision loss in the right eye during the chemotherapy. After one cycle of intravenous chemotherapy, her visual acuity decreased by 0.6 in the right eye (VOD = 0.4) compared to the previous value of 1.0 (VOD = 1.0). No evidence of ocular infiltration was observed from the cerebral magnetic resonance imaging (MRI). During her follow-up period, we documented the ophthalmologic examinations including visual acuity, visual field (VF), visual evoked potential (VEP), electroretinogram (ERG), fundus photograph (FP), fundus fluorescein angiography (FFA) and optical coherence tomography (OCT). Ophthalmoscope examination and fundus photograph showed optic disc edema, fuzzy boundary and linear hemorrhages in her right eye. Fundus fluorescein angiography (FFA) revealed capillary underdevelopment at the nasal and superior temporal area of the optic disc in the early phase and capillary fluorescein leakage in the late phase. The result of VEP test suggested the impaired function of the optic nerve. Thus, a diagnosis of nonarteritic anterior ischemic optic neuropathy (NAION) was made by the ophthalmologist according to these results. The patient was prescribed prednisone combined with neuroprotective drugs, which did not work. After the cessation of chemotherapy, her impaired vision gradually recovered.

**Conclusions:**

This is the first reported case of acute visual impairment in a patient who underwent chemotherapy of a normal dose. It is indicated that while receiving benefits from chemotherapy, cancer patients simultaneously suffer from the risk of vision loss.

## Background

Patients with malignancies benefit from the advancement in chemotherapeutic treatments, but chemotherapeutic agents also present a wide spectrum of toxic effects in parallel. In recent years, it has been reported that chemotherapy could induce irreversible or reversible visual loss due to an infrequent form of chemotherapeutic toxicity. For instance, a case that a male patient who had lung cancer suffered from acute unilateral blindness after 5 cycles of cisplatin/gemcitabine (cisplatin 80 mg/m^2^, gemcitabine 1250 mg/m^2^) was reported in a study [1]. Visual complications including visual loss are very likely to emerge after patients receive chemotherapy, which are imperative to consult at the ophthalmology department.

## Case presentation

A 48-year-old woman developed tinnitus and enlarged cervical lymph nodes. Nasopharyngoscopy examination and biopsy confirmed the diagnosis of nasopharyngeal squamous cell carcinoma, thus, she received the anti-tumor therapy of a combination of docetaxel, cisplatin and fluorouracil (TPF: docetaxel used on the first day at 75 mg/m^2^, cisplatin used on the first and second day at 75 mg/m^2^ and fluorouracil used from the first day to fifth day at 500 mg/m^2^) which was repeated every four weeks. The patient complained of visual loss in the right eye two weeks after the first cycle of chemotherapy, and she received the second cycle of chemotherapy as planned.

**Fig. 1:**
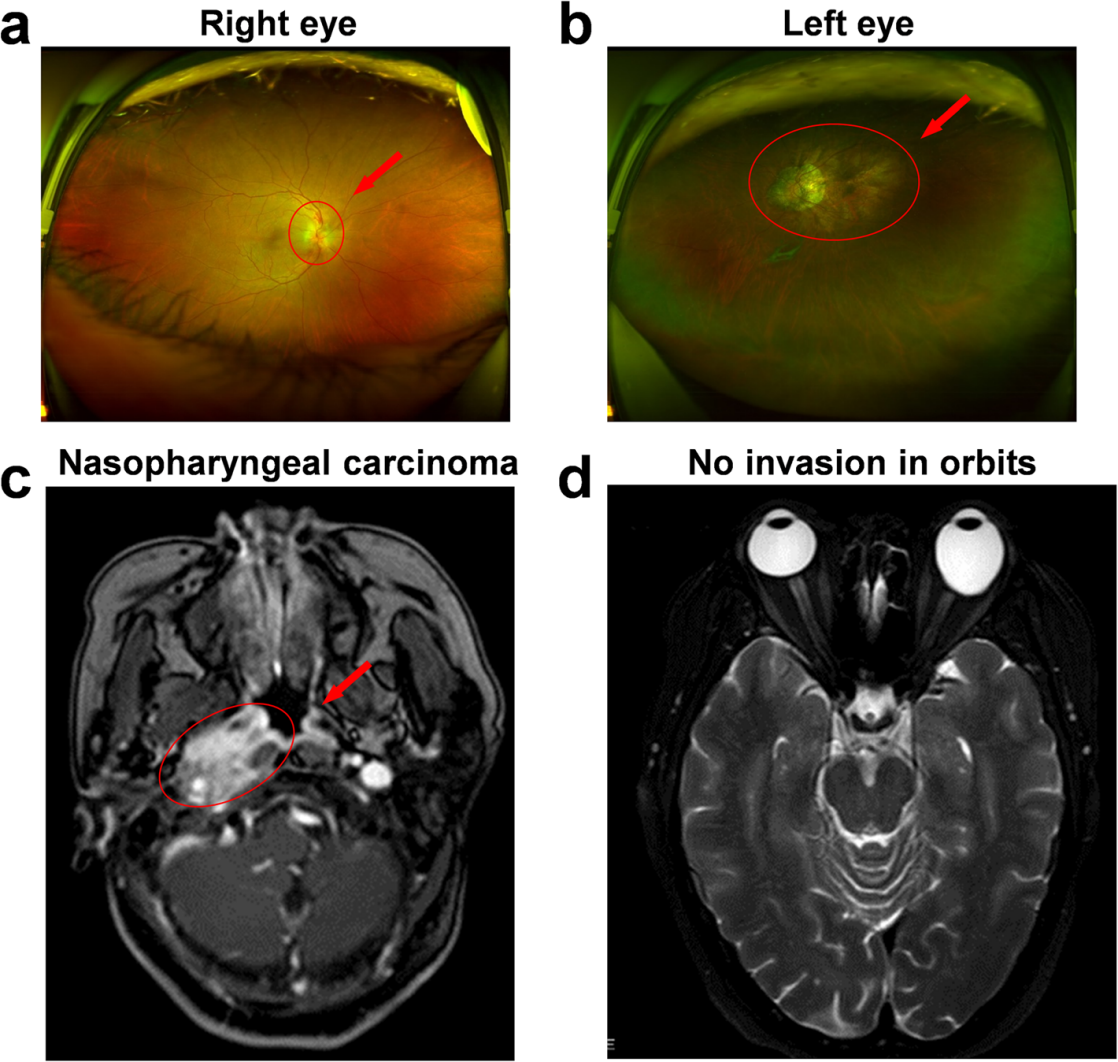
Ophthalmoscope examination and MRI images of orbits during chemotherapy. **a**: fundus photo of the right eye, **b**: fundus photo of the left eye (December 6th, 2017), **c**: MRI images of skull base invasion in nasopharyngeal carcinoma, **d**: MRI images of orbits (December 26th, 2017)

One week later, she visited an ophthalmologist for the first time because of progressing vision loss, and visual acuity was tested (VOD: visio oculus dexter, for the right eye; VOS: visio oculus sinistra, for the left eye). Her visual acuity had a decrease of 0.6 in the right eye (VOD = 0.4) compared to the original value (VOD = 1.0), and the relative afferent pupillary defect (RAPD) was tested for the right eye. She had a history of high myopia and amblyopia in her left eye evidenced by the low visual acuity (VOS=CF/25 cm, meaning that she could only count fingers at the distance of 25 cm ahead). The current vision impairment in her right eye therefore seriously affected her quality of life. Ophthalmoscope examination and fundus photograph showed optic disc edema, fuzzy boundary and linear hemorrhages in the right eye (Fig. 1a). Typical fundus changes of high myopia were observed in the left eye, such as atrophic arc around optic disc, fuchs spot and tigroid fundus (Fig. 1b). The cerebral magnetic resonance imaging (MRI) provided evidence of no tumor involvement in orbits and central visual pathways (Fig. 1c and d). The long optic axis of the left eye is a typical feature of high myopia eyeball (Fig. 1d). The patient received neurotrophic drug treatments including mecobalamine and citicoline. Three weeks later, the visual acuity in the right eye decreased to 0.3 and the signs of optic disc mentioned above also existed.

**Fig. 2:**
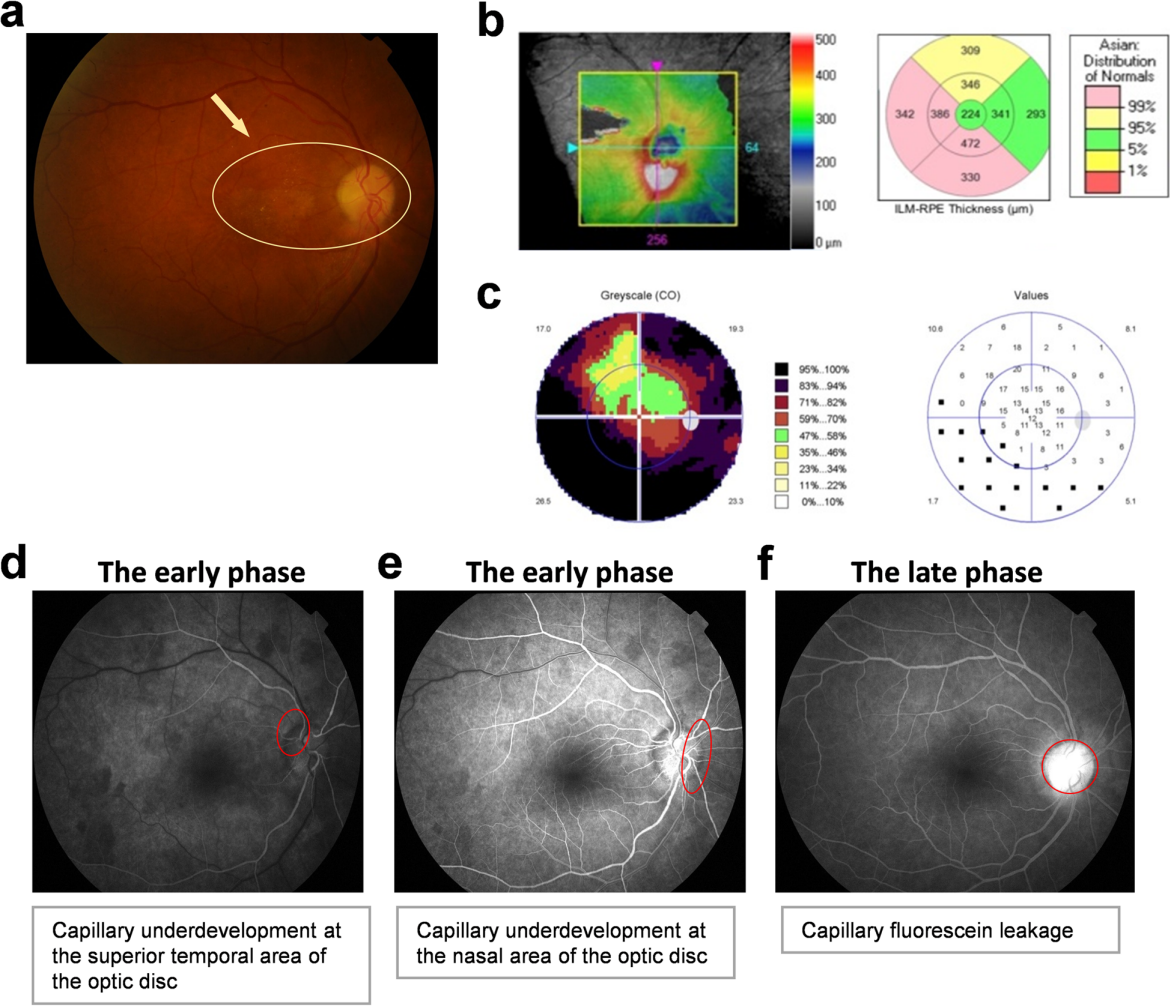
Ophthalmologic examinations of the right eye one week after chemotherapy ends. **a**: ophthalmoscope examination, **b**: optic disc OCT, **c**: visual field examination, **d**-**f**: fundus fluorescein angiography (January 5th, 2018)

About one week after the third cycle of chemotherapy, she visited the ophthalmologist for the third time. The VOD was determined to be 0.3. The optic disc edema subsided, the upper part of the optic disc became gray and residual retinal hemorrhage was located on the inferior rim. Macular pucker, depigmented macules and hard exudate were observed in the macular area (Fig. 2a). The optic disc optical coherence tomography (OCT) of the right eye showed that the thickness from the internal limiting membrane to the retinal pigment epithelium (ILM-RPE) at the superior side and nasal side was thinner than that in normal eyes (Fig. 2b). Visual field (VF) examination of the right eye indicated severe visual field defects (Fig. 2c). Fundus fluorescein angiography (FFA) as the most important assistant examination revealed an early phase capillary underdevelopment at the nasal and superior temporal area of the optic disc and a late phase capillary fluorescein leakage (Fig. 2d-f). Thus, a diagnosis of NAION was made for the right eye, and gradually reduced high-dose oral prednisone along with neuroprotective drugs were used for treatment. Meanwhile, retrobulbar injection of compound betamethasone (1 ml) combined with eye drops of ocular hypotensive agents were given to the right eye.

**Fig. 3:**
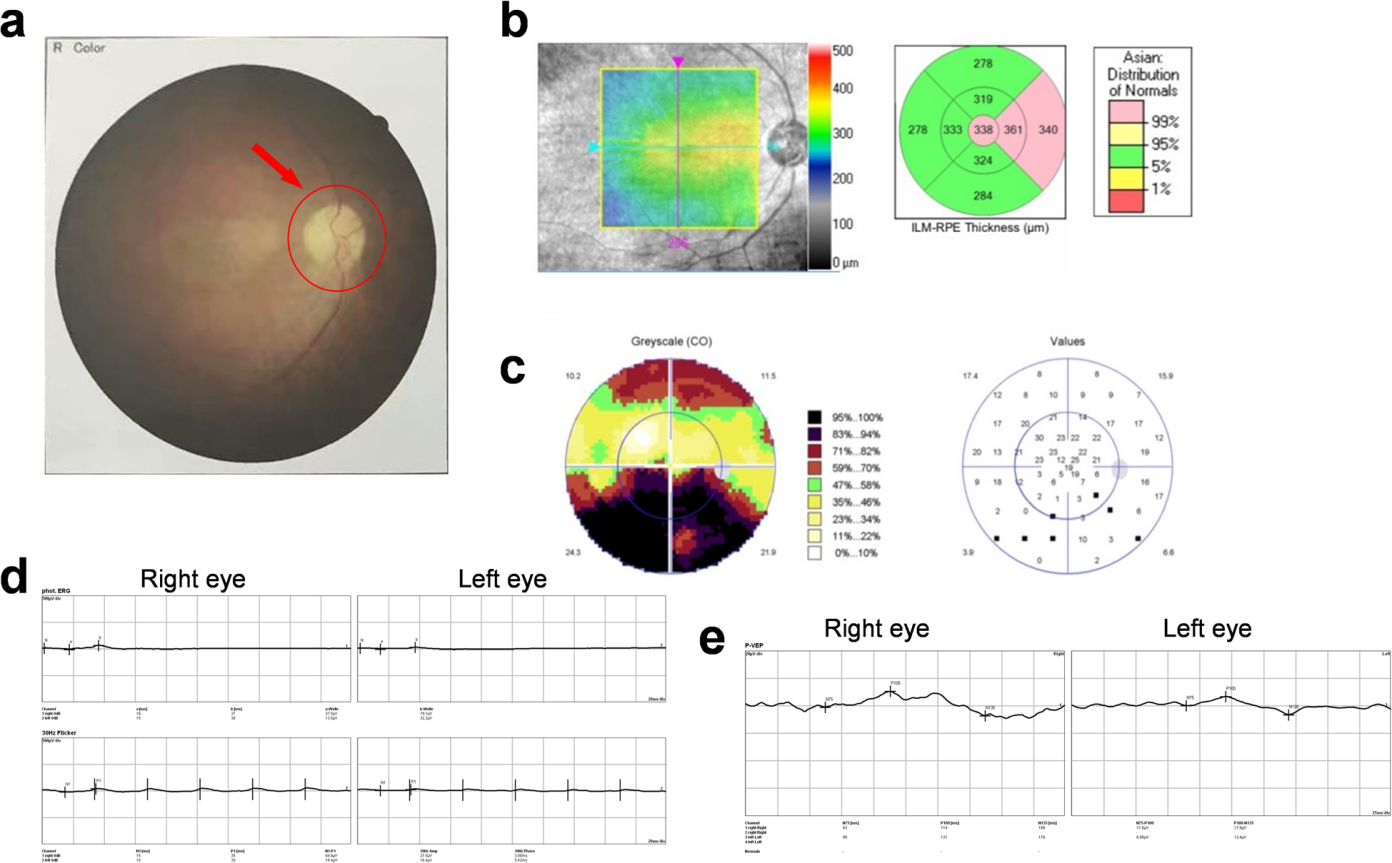
Ophthalmologic examinations of the right eye one month after chemotherapy ends. **a**: ophthalmoscope examination, **b**: macula OCT, **c**: visual field examination, **d**: ERG test, **e**: P-VEP test (January 25th, 2018)

One month after finishing three cycles of induction chemotherapy, concomitant intravenous antitumor therapy with radiotherapy was started. Due to the suspicious ocular toxicities of cisplatin, Nimotuzumab, a targeted agent, was recommended as an alternative treatment. The patient made the fourth visit to the ophthalmologist, and the VOD of the right eye was determined to be 0.4. The optic disc edema subsided with clear boundary, but the color of the optic disc was still gray (Fig. 3a). OCT showed the secondary macular epiretinal membrane (Fig. 3b). The VF improved obviously compared with the image three weeks ago, which means the function of the optic nerve had been partially repaired (Fig. 3c). The ERG results of the right eye showed that the amplitudes of a-type and b-type wave, as well as P1-type wave were almost normal, suggesting the normal function of the retina (Fig. 3d). The incubation period of P100-type wave in P-VEP test was lengthened, indicating that the function of optic nerve was partially impaired (Fig. 3e).

**Fig. 4:**
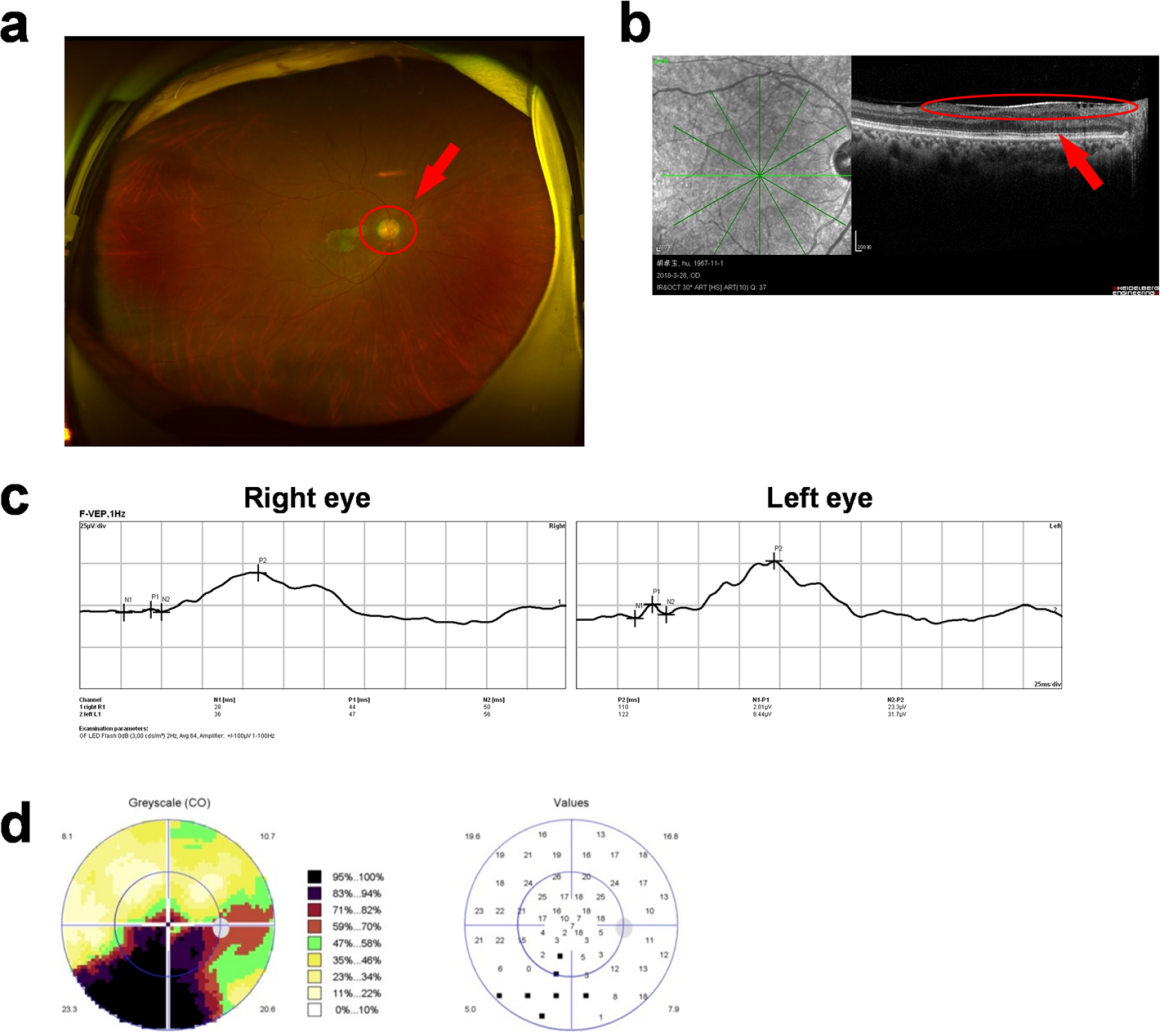
Ophthalmologic examinations of the right eye at follow-up visits to ophthalmology. **a**: ophthalmoscope examination, **b**: macula OCT, **c**: F-VEP (March 28th,2018), **d**: visual field examination (August 13th, 2018)

After finishing radiotherapy, the patient had not been treated with chemotherapy for 3 months. She made her follow-up visit at the ophthalmic clinic and the VOD improved to be 0.5. Moreover, the color of the optic disc was not as gray as before while the macular epiretinal membrane still existed (Fig. 4a and b). The amplitude and incubation periods of P2-type wave from the right eye were almost normal in F-VEP-1HZ test, which signified that the function of the optic nerve had been mostly repaired (Fig. 4c). About 7.5 months after chemotherapy, the patient’s visual acuity remained at VOD 0.5. The VF of her right eye improved dramatically (Fig. 4d). The results of ophthalmologic examinations during her follow-up period are presented in Table 1.

**Table 1:** The results of ophthalmologic examinations in the right eye during the follow-up period

Time points	Eye symptoms	Visual field	ERG	VEP	OCT	Ophthalmoscope examination	Fundus fluorescein angiography	Visual acuity
December 6th, 2017 *(during chemotherapy)*	Progressive visual loss	–	–	–	–	Optic disc edema, fuzzy boundary and linear hemorrhages.	–	0.4
December 29th, 2017 *(during chemotherapy)*	Progressive visual loss	–	–	–	–	Optic disc edema, fuzzy boundary and linear hemorrhages.	–	0.3
January 5th, 2018 *(1 week after chemotherapy)*	Progressive visual loss	Severe visual field defects	–	–	Thinner ILM-RPE at superior side and nasal side	The upper part of the optic disc becoming gray, optic disc edema subsiding and residual retinal hemorrhage on the inferior rim; creases, depigmented macules and hard exudate in the macular area.	Capillary underdevelopment at the nasal and superior temporal area of optic disc in the early phase and capillary fluorescein leakage in the late phase	0.3
January 25th, 2018 *(1 month after chemotherapy)*	Improved vision	Obviously improved visual field.	Normal amplitudes of a-type wave, b-type wave and P1-type wave.	Lengthened incubation period of P100-type wave in P-VEP test.	Secondary macular epiretinal membrane	Gray optic disc without edema and with clear boundary.	–	0.4
March 28th, 2018 *(3 months after chemotherapy)*	Improved vision	–	–	Normal incubation period of P2-type wave.	Secondary macular epiretinal membrane.	The optic disc becoming not so gray as before.	–	0.5
August 13th, 2018 *(7.5 months after chemotherapy)*	Improved vision	Dramatically improved visual field.	–	–	–	–	–	0.5

Note: On October 31st, 2017 the patient received the first cycle of chemotherapy. On November 30th, 2017, she received the second cycle of chemotherapy. On December 29th, 2017, she received the third cycle of chemotherapy. From January 26th, 2018 to March 22th, 2018, she received radiotherapy combined with targeted therapy. *VEP* visual evoked potential, *ERG* electroretinogram, *OCT* optical coherence tomography

## Discussion and conclusions

The neck mass constitutes the most common presenting symptom of nasopharyngeal carcinoma (NPC) [2]. Impaired vision as the initial presentation of NPC caused by the involvement of the optic nerve was rarely reported. As a treatment of NPC, neoadjuvant chemotherapy was currently reported to effectively reduce local-regional recurrences and distant metastases [3]. Fluorouracil and cisplatin are commonly used with major toxicities including myelo-suppression and vomiting. Ocular complications as rare toxicities have not been widely recognized yet and are difficult to be detected. Owing to the improvement in anti-cancer therapies, NPC manifests a favorable prognosis, which helps patients to live a longer life. In addition, healthy eyes and good vision represent an important part of life quality. Therefore, we reported a case aiming at raising the attention to chemotherapy-induced ocular toxicity in the treatment of NPC.

Due to a sharp decrease of visual acuity, the patient was suggested to consult in an ophthalmology clinic for etiological diagnosis and potential therapy. The diagnoses of glaucoma, cataracts, macular degeneration and other eye diseases were excluded; the right optic disk edema was observed by funduscopic examination; and a diagnosis of NAION was made with etiology unknown. NAION is the most common cause of acute visual loss in people aged over 50, resulting from non-inflammatory small vessel ischemic damage to the anterior portion of the optic nerve. However, the cause and pathogenesis of this disorder remain unclear [4, 5]. Neuroprotective drugs or agents acting on the disc edema are often included in the treatment of NAION, but currently none of the therapy has yet been proved to be effective [6]. Given the temporal relationship between chemotherapy and vision loss of the patient, we suspected that the onset of NAION might be attributed to the intravenous chemotherapy. Because of a combination of drugs, it is difficult to identify a specific agent accounting for the followed vision loss. Since the toxic effect on the retinal or optic nerve might cause irreversible vision loss, the early detection of ocular toxicity and the opportune cessation of anti-cancer therapy are necessary. However, sometimes a trade-off between the risk of permanent visual damage and the effectiveness of anti-cancer therapy may emerge.

Vision loss attributed to cisplatin, docetaxel and fluorine has been reported in several previous studies. Cisplatin-associated retinal toxicity including blurred vision, color vision defects, and electroretinographic (ERG) changes was dose-dependent or unique to high doses. There was a case that a patient with lung cancer who received five cycles of cisplatin/gemcitabine treatment for lung cancer was admitted to the emergency room unfortunately and complained of acute blindness in his left eye. His fundus examination was normal in both eyes, and the MRI of the left optic nerve and orbit did not reveal any relevant findings. A diagnosis of left retrobulbar optic neuritis was made eventually [1]. There was another case that a 55-year-old man planned to receive a 4-day continuous infusion of cisplatin at a dose of 25 mg/m^2^ daily as part of a chemotherapeutic salvage regimen for non-Hodgkin lymphoma, but received actual dose of 100 mg/m^2^ daily for 4 days inadvertently. Except for anorexia, nausea and tinnitus, he developed bilateral decreased vision immediately after the treatment. The ERG showed diminished a-wave and missed b-wave [7]. A clinical study of 52 patients determined the prevalence rates of 5-FU-associated ocular abnormalities [8]. The results showed that the most common presentation was tearing (26.9%), followed by blurred vision (11.5%). After receiving 12 courses of intravenous 5-FU for metastatic breast cancer, a 72-year-old woman complained of a sudden visual loss, and her vision started to recover after the discontinuation of anti-cancer agent. A deficiency of dihydropyrimidine dehydrogenase (DPD) was detected and 5-FU was considered to be responsible for the visual loss associated with DPD deficiency [9]. Another female who received monthly intravenous infusion of docetaxel for metastastic breast cancer also complained of blurred vision in both eyes two months later after the treatment. Then docetaxel was replaced by Xeloda, and her vision started to recover [10].

When patients suddenly develop vision loss while receiving chemotherapy, the possibility of chemotherapy-induced NAION should be considered. Most ophthalmic complications are reversible if recognized in an early phase. Besides, dosage reduction or agent cessation could rescue patients from vision loss. However, if the optic nerve or the retinal was involved, patients might develop irreversible vision loss. Induction chemotherapy plays a crucial role in the treatment of local advanced nasopharyngeal carcinoma, and presented here was one case of severe visual impairment induced by chemotherapy. Therefore, it is indicated that cancer patients take the risk of vision loss while benefiting from chemotherapy.

Although it has been reported that intravenous chemotherapy of cisplatin and docetaxel could cause retinal toxicity, this is the first case demonstrating that intravenous administration of chemotherapy (TPF) for NPC could induce NAION and cause irreversible vision loss. This reported case suggests that when patients suddenly develop visual impairment after receiving chemotherapy, the possibility of ophthalmic complications should be considered. In addition, as soon as symptoms are recognized, the patient should be scheduled for further examinations in the ophthalmology clinic, and a cooperation between oncologists and ophthalmologists is necessary for subsequent treatment.

## Data Availability

The data supporting the conclusions of this article are included within the article.

## Abbreviations

ERG: Electroretinogram
FFA: Fundus fluorescein angiography
FP: Fundus photograph
ILM: Internal limiting membrane
MRI: Magnetic resonance imaging
NAION: Nonarteritic anterior ischemic optic neuropathy
NPC: Nasopharyngeal carcinoma
OCT: Optical coherence tomography
RAPD: Relative afferent pupillary defect
VEP: Visual evoked potential
VF: Visual field
VOD: Visio oculus dexter, for the right eye
VOS: Visio oculus sinistra
